# Programmed Chromosome Deletion in the Ciliate *Oxytricha trifallax*

**DOI:** 10.1534/g3.118.200930

**Published:** 2019-10-14

**Authors:** Derek M. Clay, V. Talya Yerlici, Danylo J. Villano, Laura F. Landweber

**Affiliations:** * Departments of Biochemistry and Molecular Biophysics and Biological Sciences, Columbia University, New York, NY and; † Department of Molecular Biology, Princeton University, Princeton, NJ

**Keywords:** Genomic Rearrangement, Ciliates, Epigenetics

## Abstract

The ciliate *Oxytricha trifallax* contains two nuclei: a germline micronucleus and a somatic macronucleus. These two nuclei diverge significantly in genomic structure. The micronucleus contains approximately 100 chromosomes of megabase scale, while the macronucleus contains 16,000 gene-sized, high ploidy “nanochromosomes.” During its sexual cycle, a copy of the zygotic germline micronucleus develops into a somatic macronucleus via DNA excision and rearrangement. The rearrangement process is guided by multiple RNA-based pathways that program the epigenetic inheritance of sequences in the parental macronucleus of the subsequent generation. Here, we show that the introduction of synthetic DNA molecules homologous to a complete native nanochromosome during the rearrangement process results in either loss or heavy copy number reduction of the targeted nanochromosome in the macronucleus of the subsequent generation. This phenomenon was tested on a variety of nanochromosomes with different micronuclear structures, with deletions resulting in all cases. Deletion of the targeted nanochromosome results in the loss of expression of the targeted genes, including gene knockout phenotypes that were phenocopied using alternative knockdown approaches. Further investigation of the chromosome deletion showed that, although the full length nanochromosome was lost, remnants of the targeted chromosome remain. We were also able to detect the presence of telomeres on these remnants. The chromosome deletions and remnants are epigenetically inherited when backcrossed to wild type strains, suggesting that an undiscovered mechanism programs DNA elimination and cytoplasmically transfers to both daughter cells during conjugation. Programmed deletion of targeted chromosomes provides a novel approach to investigate genome rearrangement and expands the available strategies for gene knockout in *Oxytricha trifallax*.

The dual nuclear structure of the ciliate *Oxytricha* trifallax partitions the germline and somatic functional elements into two distinct nuclei, the micronucleus (MIC) and macronucleus (MAC). The somatic macronucleus is comprised of approximately 16,000 gene-sized chromosomes, while the germline micronucleus contains on the order of 100 megabase-sized chromosomes (Swart *et al.* 2013; Chen et al. 2014). Under normal growth conditions, like other ciliates, *Oxytricha* reproduces asexually and its macronuclear nanochromosomes are maintained through amitotic division at an average of 2,000 copies per cell (Prescott 1994). Under stressful conditions, the cell can undergo a non-reproductive sexual cycle. During *Oxytricha’s* sexual development, cells of different mating types pair and transfer a single post-meiotic haploid micronucleus. The exchanged haploid nuclei fuse with their non-exchanged counterpart to form a new diploid micronucleus. The new macronucleus develops from a copy of the newly formed micronucleus through elimination of more than 90% of the DNA sequence (Chen *et al.* 2014) and rearrangement of the remaining sequences generates nanochromosomes in the new macronucleus (reviewed in Yerlici and Landweber 2014).

Ciliates have specific mechanisms to identify sequences for retention or deletion from the rearranging nucleus. In the distantly related ciliates *Tetrahymena* and *Paramecium*, the differentiation of germline-limited internally eliminated sequences (IESs) from macronuclear destined sequences (MDSs) is programmed by scan RNAs (scnRNA) (reviewed in Allen and Nowacki 2017). These small RNAs are produced from the parental micronucleus during genomic rearrangement and are selectively enriched by alignment to macronuclear sequences. Small RNAs that do not match sequences in the parental macronucleus proceed to mark sequences in the developing macronucleus for excision and elimination.

In *Paramecium* species, there is evidence of an interaction between the scnRNA and RNA interference (RNAi) pathways that can reprogram MDS deletion in subsequent generation. Experimental introduction of truncated genes into the vegetative macronucleus leads to the production of siRNA against the targeted gene and the subsequent silencing of both the native and introduced copies of the gene (Meyer 1992, Ruiz *et al.* 1998, Galvani and Sperling 2001, Garnier *et al.* 2004, Götz *et al.* 2016). Upon undergoing conjugation, this RNAi silencing can trigger, in the subsequent generation, deletion of the genomic regions, flanked by a “TA” dinucleotide, which is the cut site for PiggyMAC transposases (Klobutcher and Herrick 1995; Baudry *et al.* 2009). Further evidence of this interaction came from the observation that injection of dsRNA against specific MDS regions resulted in the deletion of these MDSs from the next generation’s macronucleus (Garnier *et al.* 2004).

In contrast to *Paramecium* and *Tetrahymena*, the RNA pathways reported so far in *Oxytricha* mark sequences for retention rather than deletion. Early in *Oxytricha*’s genomic rearrangement process, the parental macronuclear genome appears to be completely transcribed, generating long RNA templates (Nowacki *et al.* 2008; Lindblad *et al.* 2017) together with the production of 27 nucleotide piRNAs (Fang *et al.* 2012; Zahler *et al.* 2012). Collectively, these template RNAs and piRNAs mark retained sequences and program the necessary rearrangements to convert a germline into the somatic nucleus. Experimentally, injection of synthetic piRNAs can result in the retention of normally deleted IESs in the macronucleus. In *Oxytricha*, there is no simple consensus dinucleotide “TA” cut site. Instead, adjacent MDS ends in the macronucleus contain microhomologous sequences that vary and can be much longer (Chen *et al.* 2014). Only one copy of the microhomologous sequence is retained in the macronucleus after MDSs fuse during rearrangement. The general presence of these microhomologous repeats at the ends of MDSs has long suggested that they play an important role in facilitating *Oxytricha*’s genomic rearrangement (Prescott 1994).

Introduction of artificial DNA or RNA templates early in genomic rearrangement reprograms rearrangement of the next generation’s macronucleus (Nowacki *et al.* 2008, Fang *et al.* 2012). Here, we show that introduction of synthetic copies of a full length nanochromosome early in genomic rearrangement can reprogram deletion of that chromosome from the subsequent generation’s macronucleus. Furthermore, we exploited this surprising observation to develop a tool for targeted chromosomal deletion to facilitate functional genetic manipulation of *Oxytricha trifallax*.

## Methods

### Generation of DNA constructs for microinjection

To generate full length DNA templates including small (30 to 50 bp) deletions, overlap extension PCR was performed using JRB310 genomic DNA as template and Phusion DNA Polymerase (NEB). PCR Primers were synthesized by IDT with standard desalting conditions. Wild type DNA constructs were produced via conventional PCR with JRB310 genomic DNA as template. The PCR products were gel purified using Qiagen MinElute columns according to manufacturer’s instructions. PCR products were singly A-tailed with Taq polymerase (Roche) and TA-TOPO cloned (Invitrogen). TOPO cloned plasmids were transformed into TOP10 One shot chemically competent cells (Invitrogen) according to manufacturer’s instructions. Plasmid DNA was isolated from individual clones using the QIAprep Spin Miniprep kit (Qiagen). TOPO plasmids were verified via Sanger sequencing through Genewiz.

Validated plasmids were used as templates for PCR to generate approximately 100 μg of PCR product. Quality of the PCR products was verified by gel electrophoresis. PCR products were phenol:chloroform extracted and concentrated by ethanol precipitation. DNA pellets were resuspended in nuclease-free water (Ambion) and run through an ultra-free MC column (Millipore) according to manufacturer’s instructions. DNA was brought to a final concentration of 1 to 3 mg/mL for microinjection.

### Oxytricha culturing

*Oxytricha trifallax* cells were grown in Pringsheim media (0.11 mM Na_2_HPO_4_, 0.08mM MgSO_4_, 0.85 mM Ca(NO_3_)_2_, 0.35 mM KCl, pH 7.0), fed *Chlamydomonas reinhardtii* and supplemented with *Klebsiella* for improved growth. Cells were cleansed of debris and algae by filtering through cheesecloth and concentrated via centrifugation at 80g for 1 min for harvesting (Khurana *et al.*, 2014).

### DNA template injections and screening for deletion lines

*Oxytricha* cells were mated approximately 1 to 4 weeks post-excystment by mixing 3 mL of each mating type, JRB310 and JRB510, along with 6 mL of fresh 1X Pringsheim. At 3 to 5 hr post mixing, pairs were isolated and placed in Volvic water with 0.2% bovine serum albumin according to previously published methods (Fang *et al.* 2012). DNA molecules were injected at 3 to 5 hr post-mixing into the macronuclei of the paired cells as previously described except for the timing (Nowacki *et al.* 2008). After injection cells were pooled in Volvic water to improve survival rates. At 60 to 72 hr post mixing, the pooled cells were singled out to grow clonal injected cell lines. As clonal population size grew, lines were transferred to 10 cm petri dishes and grown in 1X Pringsheim media.

*Oxytricha* clonal lines were screened for deletions using cells or purified genomic DNA in PCR. Genomic DNA was harvested via the NucleoSpin Tissue kit (Macherey-Nagel). Cells were concentrated via centrifugation for 1 min at 80g, and the supernatant was aspirated.

### Mating of deletion lines

Using the same method for setting up mating as above, pairs were collected at 6 to 12 hr post mixing and pooled. Clonal lines were isolated at 60 to 72 hr post mixing and cultured according to the protocol described above.

### RNA isolation and cDNA synthesis

RNA was collected from pools of isolated pairs (approximately 50 pairs) at 12 hr post-mixing via the mirVANA miRNA Isolation Kit (Ambion) using the total RNA isolation protocol according to manufacturer’s instructions. RNA was DNAse-treated with Turbo DNAse (Ambion) according to the manufacturer’s instructions. SuperScript III First-Strand Synthesis (Thermofisher Scientific) kit was used according to manufacturer’s instructions to generate cDNA for qPCR analysis.

### Quantitative PCR for genomic DNA and cDNA

Quantitative PCR was done with Sybr green power mix (ABI) according to the manufacturer’s instructions using either ABI 7900 (ABI) or CFX384 (BioRad) qPCR machines. Standards were generated via conventional PCR and purified by MinElute PCR purification (Qiagen). Purified standards were quantified by Qubit High Sensitivity DNA Assay Kit following the standard protocol (Thermofisher Scientific). Results from qPCR were quantitated via ΔΔCT and standard curves.

### Southern Hybridization

200 ng of genomic DNA was loaded onto a 1.0% agarose gel and Southern transfer and hybridization were performed according to previously published protocols (Bracht *et al.* 2017) with minor alterations.

### Illumina sequencing of deletion lines

Genomic DNA from deletion and wild type cell lines were used for preparing TruSeq Illumina libraries according to manufacturer’s instructions and sequenced in single-end mode on the Illumina HiSeq2500 platform. Raw reads (average length 150nt) were processed using Galaxy on the Princeton University webserver (galaxy.princeton.edu) (Goecks *et al.* 2010). Reads were trimmed using Trim Galore version 0.4.3 to remove low quality ends (<Q20) and adapter sequences (maximum allowed error rat: 0.1, minimum read length: 20nt) (Felix Krueger, Babraham Institute https://www.bioinformatics.babraham.ac.uk/projects/trim_galore/). Duplicate reads were collapsed using FASTX-toolkit (Assaf Gordon, http://hannonlab.cshl.edu/fastx_toolkit/).

Reads were then mapped using BWA-mem with default parameters (Li and Durbin 2009) onto the *Oxytricha* JRB310 MAC genome assembly (Lindblad *et al.* 2019). SAM files were processed to remove alignments with a low mapping score and non-primary alignments using SAMtools view (parameters: -bSq 5 and -F 256) (Li *et al.* 2009). The mapped reads from each library were then subsampled using seqtk (https://github.com/lh3/seqtk) according to the number of reads mapping to the macronuclear genome to normalize for sequencing depth and converted into bedgraph format using BEDtools genomecov (Quinlan and Hall 2010). Coverage tracks were generated using Bioconductor software Sushi package (Phanstiel 2015) (R version 3.4.1).

### Data Availability

Strains are available on request. Supplemental file 1 contains the nucleotide sequences from the Sanger sequencing. Genomic sequencing data are available in supplemental files 2 through 14. Supplemental material available at FigShare: https://doi.org/10.25387/g3.7123214.

## Results

### DNA injection can trigger chromosome deletion

**Figure 1:**
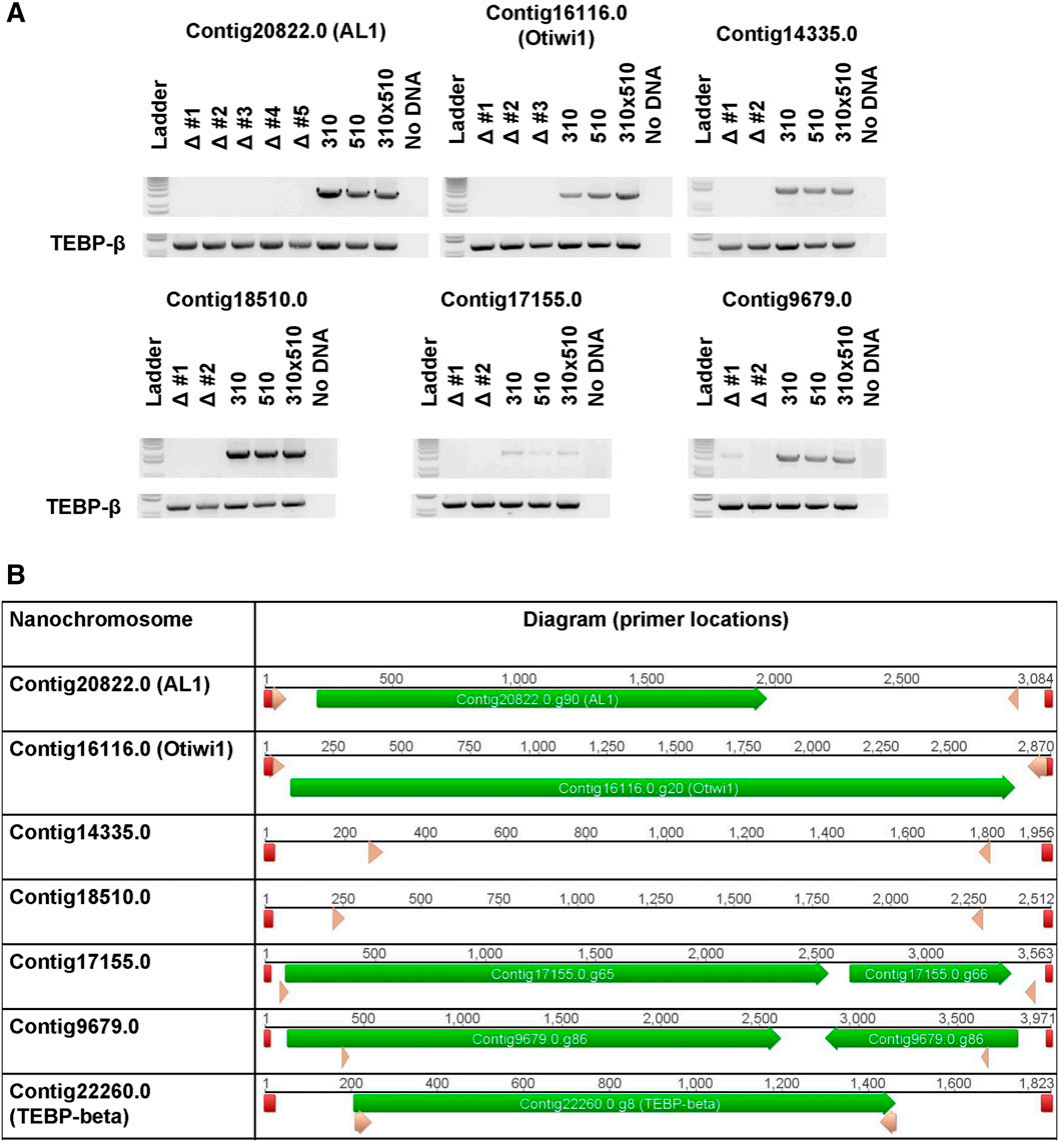
DNA injections result in the deletion of nanochromosomes from the subsequent generation. A) PCR amplification from genomic DNA harvested from screened and established lines shows loss of the targeted nanochromosome. The top gel section of each panel shows the deleted chromosome as labeled above, with the name of deletion lines (marked Δ) established and assayed in this experiment. The bottom gel section of each panel shows PCR amplification of the TEBP-β gene as input loading control. Deletion line shown in comparison to the parental lines (strains JRB310 and JRB510, marked simply 310 or 510) or the uninjected F1 population derived from mating the two parental lines. In the ΔOtiwi1 panels, deletion lines 1 and 2 were generated through injection of a construct containing a 28 bp deletion while line 3 was generated using a full length Otiwi1 chromosome construct. B) Diagram of the nanochromosomes in the PCRs in panel A with the following features labeled: telomeres (red), the locations of genes (green), and the location of the primers used in panel A (pink).

Previous research demonstrated that injection of synthetic DNA or RNA copies of chromosomes during nuclear development can specifically influence DNA rearrangement in the subsequent generation (Nowacki *et al.* 2008; Nowacki *et al.* 2010; Bracht *et al.* 2017). To further investigate the influence of exposure to a synthetic DNA molecule during conjugation, we injected a copy of wild type Contig16116.0, the nanochromosome encoding the Otiwi1 gene, into *O. trifallax* cells. Surprisingly, some (approximately 10%) of the resulting progeny displayed significant copy number reduction of the endogenous chromosome (Figure 1). In addition, we injected a modified version of Contig16116.0, containing a 28 nucleotide deletion flanked by 7 base pair endogenous repeats (see supplementary figure 1), anticipating that this DNA template could program deletion of the 28 nt sequence in the next generation’s somatic genome (Nowacki *et al.* 2008). Instead, we observed deletion of the endogenous chromosome, suggesting robustness of this effect to modest deletions in the injected template (Figure 1).

**Figure 2:**
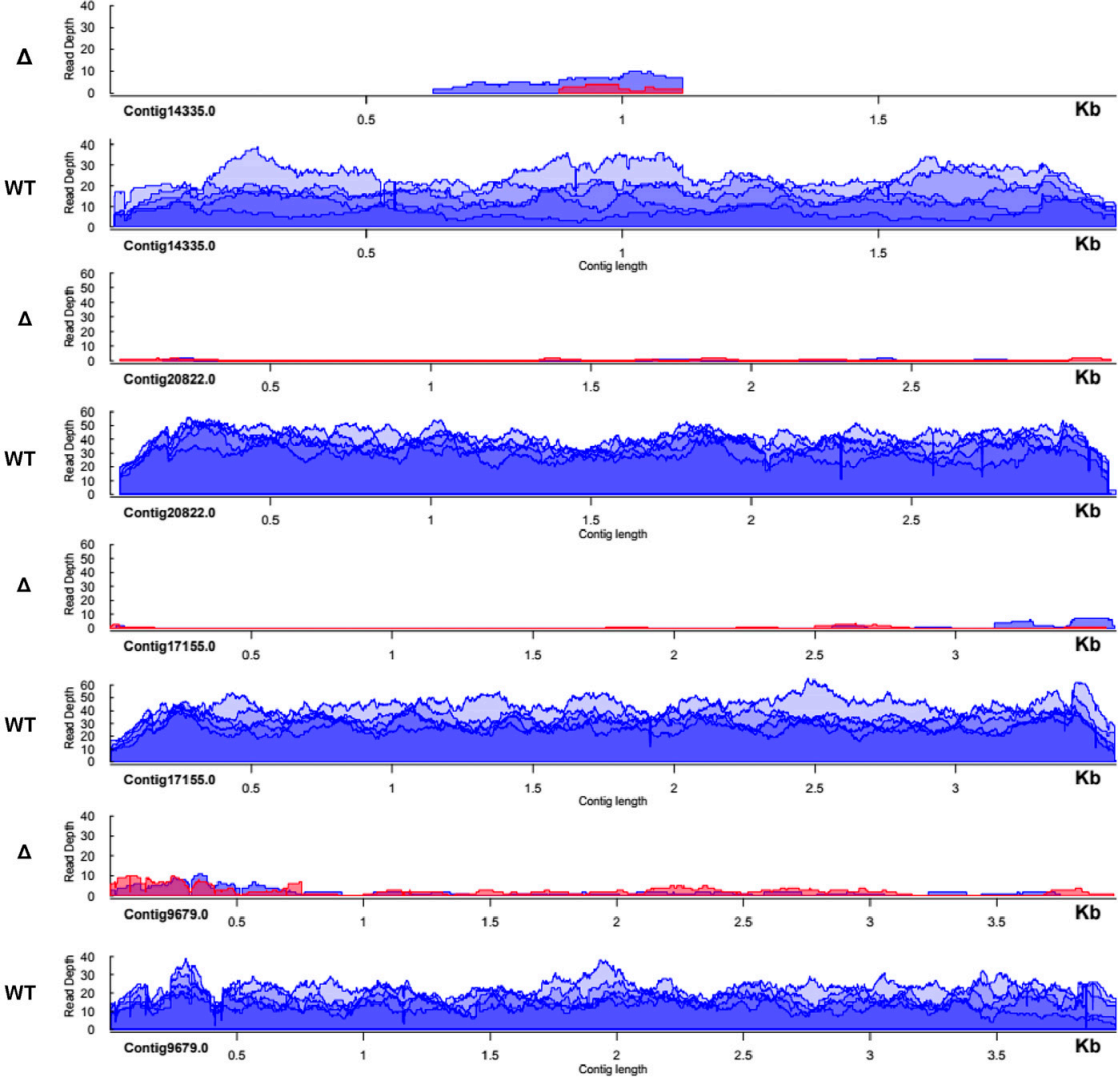
Sequence coverage for a deleted chromosome is decreased in deletion lines. Normalized read depth from Illumina whole genome sequencing mapped to targeted chromosomes shows a strong decrease in the deletion lines relative to the uninjected F1 lines derived from JRB310xJRB510 matings. The first deletion line is labeled in red and second line depicted in blue.

In order to determine the extent of this phenomenon; five additional chromosomes were tested. Contig20822.0 was also injected as a full-length DNA chromosome containing a 37 nucleotide deletion, whereas the remaining four cases were injected as full-length wild type DNA versions (Figure 1 and supplementary figure 1). qPCR analysis and whole genome sequencing demonstrated severe copy number reduction of the target chromosome within the established cell lines, with some variation in DNA copy level at regions across the chromosomes (Figure 2 and supplementary figure 2).

### Some cases of chromosome deletion result in maintenance of DNA fragments

**Figure 3:**
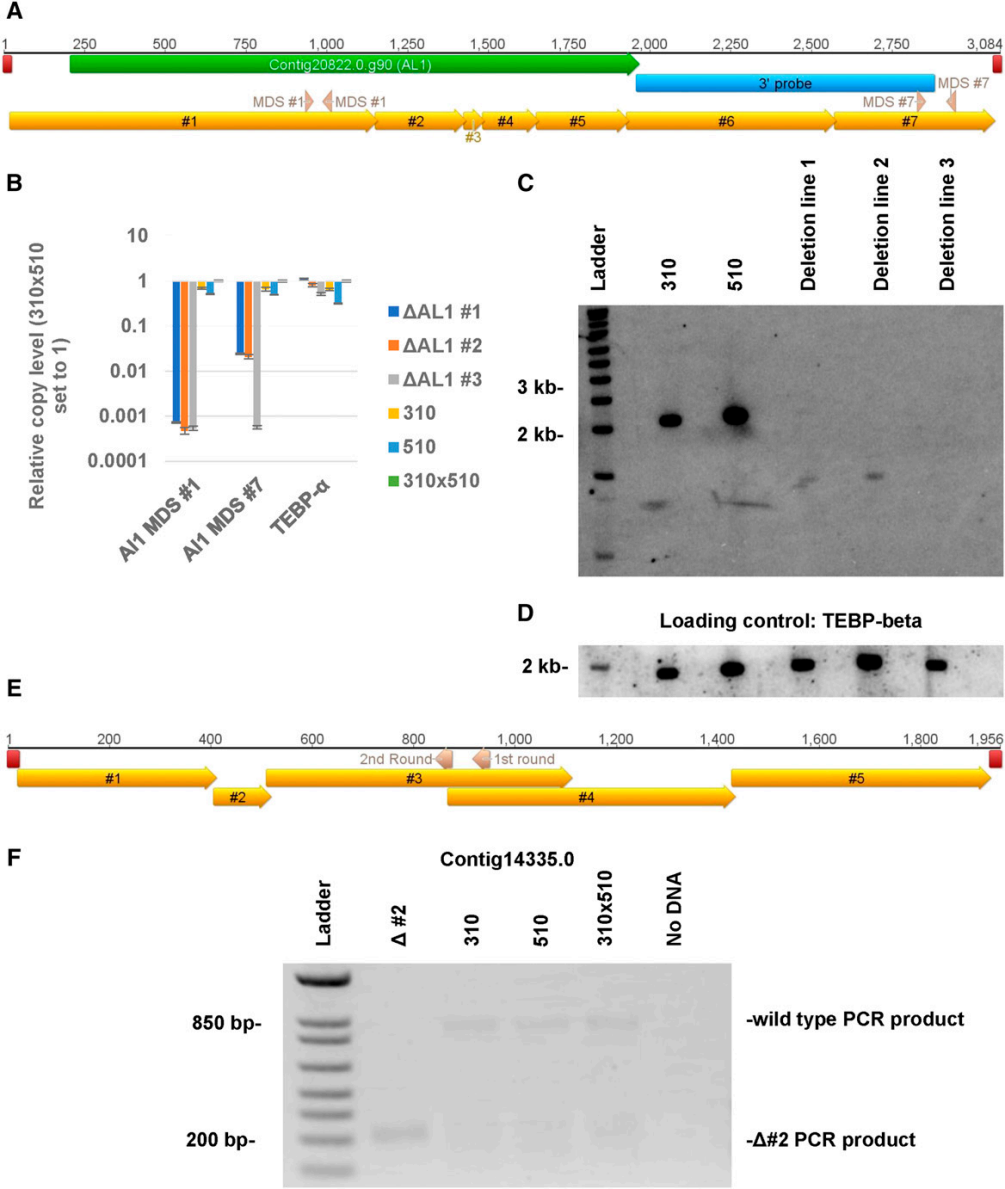
Remnants of deleted chromosomes are detected in some of the deletion lines. A) A diagram of the AL1 nanochromosome with various features labeled, including the locations of the telomeres (red), the AL1 gene (green), the MDSs (gold), the qPCR primers (pink, labeled with which qPCR they belong to in panel B, and hybridization region of the Southern probe (blue). B) Quantification of the 3′ and 5′ end of the chromosome via qPCR and the relative copy *vs.* the JRB310xJRB510 F1 genomic DNA. Error bars represent the standard deviation. C) Southern analysis of the first 3 contig20822.0 (AL1) deletion lines (#1, #2, and #3) using a probe against the 3′ end of the nanochromosome. D) The same membrane was then stripped and re-probed with TEBP-beta for loading control. E) A depiction of the contig14335.0 nanochromosome with MDSs (gold), telomeres (red), and the gene specific primers for the two rounds of telomeric PCR (pink, labeled with which PCR round they are involved in) labeled. F) Second round of telomeric PCR (see methods for description of telomeric PCR) for the 5′ end of the Contig14335.0 chromosome in contig14335.0 deletion line #2. Telomeric PCR products from contig14335.0 deletion line #2 were Sanger sequenced to identify the exact location of the aberrant telomere addition (see supplementary figure 6).

To compare copy number variation across different locations on the same chromosome, including the 5′ and 3′ ends of Contig20822.0 that encodes an Alba domain gene (hence known as Alba-like 1 or AL1), a Southern blot was performed on these deletion lines, together with the control parental strains, against a probe for the 3′ end of the chromosome (Figure 3C). This also indicated a significant reduction in the full-length AL1 chromosome levels. Furthermore, in agreement with the qPCR results (Figure 3B), deletion lines 1 and 2 (Figure 3 panels B and C) appear to contain a less abundant, shorter 3′ chromosome fragment. Whole genome sequencing reads were used to search for the presence of short chromosome remnants in other deletion lines.

The most abundant set of remnants was identified in Contig14335.0 deletion line 2, which has high Illumina read coverage exclusively in the center of the nanochromosome, and no sequence coverage at the ends (Figure 2). Moreover, telomeric reads mapping to the central portion of Contig14335.0 were identified in the deletion lines but not the wild type controls. In order to confirm the presence of telomeres on the remnants, PCR using a telomeric primer and a gene specific primer was performed as in (Chang *et al.* 2004) on Contig14335.0, deletion line 2. This telomeric PCR confirmed the presence of an internal telomere addition site in the deletion line (Figure 3E). In the case of Contig16116.0, an aberrant product was the dominant product in the conventional PCR screening of deletion line 1 (see supplementary figure 3). Sanger sequencing of this product revealed that it contained incorrect MDS fusion sites at a series of microhomologous repeats. (sequence data provided in supplementary file 1).

### Chromosome deletion eliminates expression of genes on the nanochromosome

The ostensible loss or severe copy number reduction of the targeted nanochromosomes could lead to a phenotype since the genes present on these nanochromosomes are heavily reduced in DNA copy number or completely absent from the somatic macronucleus. Indeed, we observed the same conjugation specific lethality in the Otiwi1 (Contig16116.0) deletion lines that phenocopy knockdown of the Otiwi1 gene (Fang *et al.* 2012). In addition, RT-PCR and qPCR analysis of the AL1 (Contig20822.0) deletion lines showed a massive reduction in the expression of the AL1 gene (supplementary figure 4). Thus, programmed chromosome deletion provides a novel mechanism for generating somatic mutant strains in *O. trifallax*. Furthermore, deletion of the AL1 gene results in a lethal phenotype when the deletion lines are mated to each other, with no cells surviving beyond 30hrs post-mixing. This suggests the AL1 gene is essential during sexual development, and provides proof of principle for the use of chromosome deletion to test gene essentiality.

### Epigenetics of chromosome deletion

Given our knowledge that macronuclear chromosome architecture relies on the transfer of parental epigenetic information across sexual generations (Nowacki *et al.* 2008; Fang *et al.* 2012; Bracht *et al.* 2017), we examined the influence across generations of loss or copy number reduction of targeted nanochromosomes. Because the deletion lines for Contigs 17155.0 and 9679.0 target genes are under further investigation elsewhere (Yerlici *et al*. 2019) and Contigs 20822.0 and 16116.0 encode genes essential for conjugation, we tested the ability of the deletion lines for Contigs 14335.0 and 18510.0 to generate sexual progeny, since both of these nanochromosomes contain no predicted genes.

Contig18510.0 deletion lines can be successfully backcrossed to the parental strain JRB510. Their progeny, however, do display recovery of the deleted chromosome via conventional PCR (see supplementary figure 5C).

**Figure 4:**
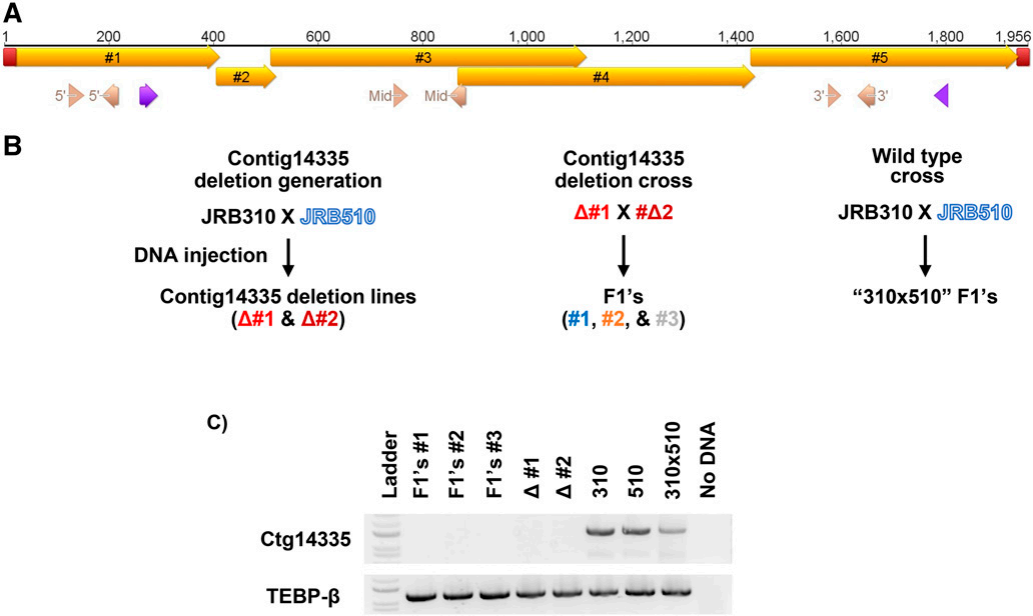

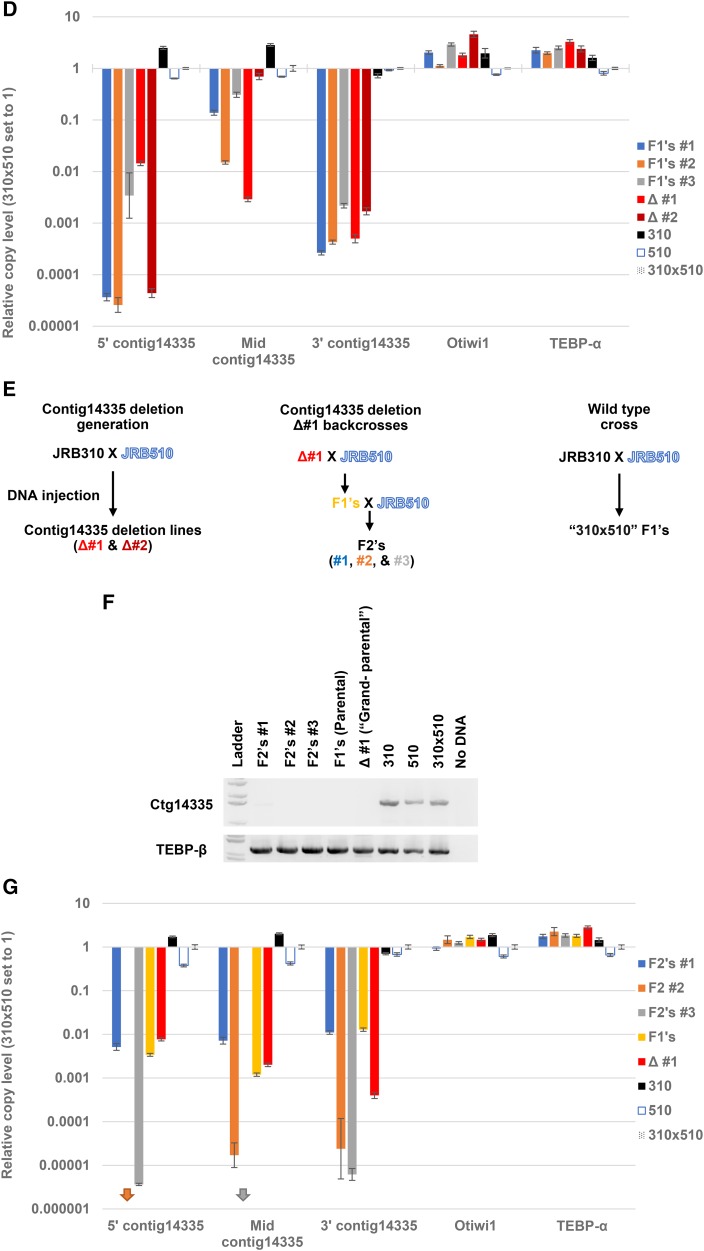

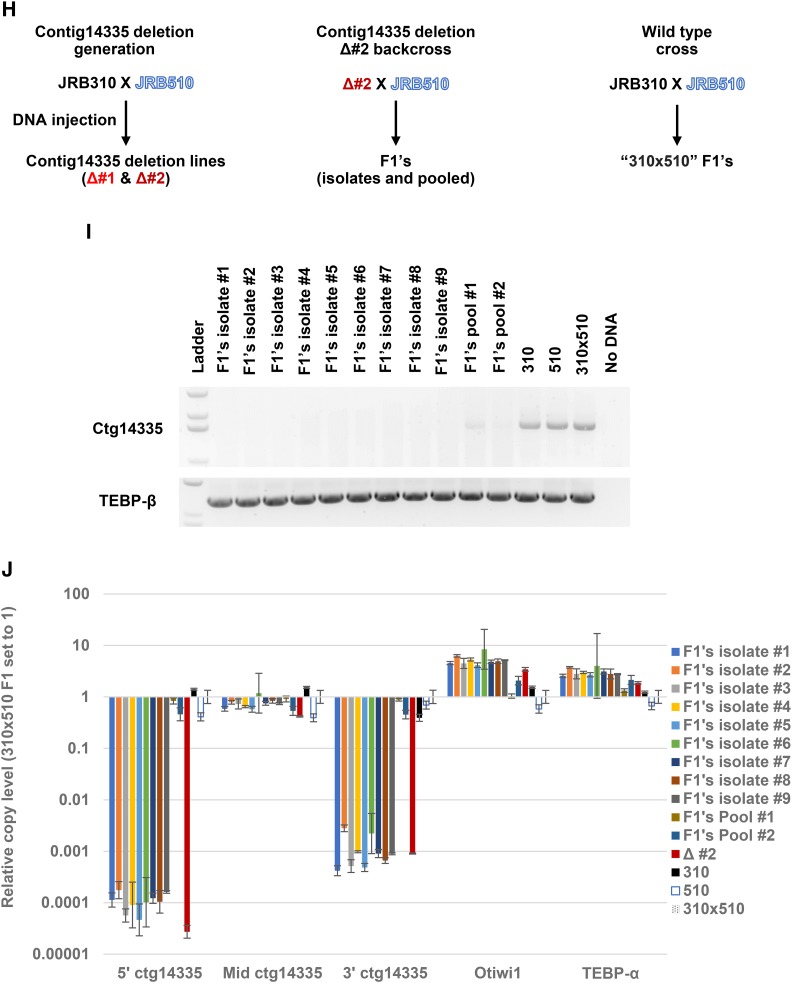
Deletions and remnants are epigenetically inherited A) An illustration of the contig14335.0 with primer locations depicted (purple for the detection PCR shown in panels C and E, pink for the qPCR primers, labeled with the corresponding qPCR they belong to in panels C, E, G). In addition, the locations of the MDSs (gold) and telomeres (red) on the nanochromosome are shown. B) Diagram of the various crosses involved generating the progeny involved in panels C and D. The colors in the diagram correspond to the colors in panel D. C) PCR amplification of genomic DNA harvested from populations of the progeny of ctg14335.0 deletion line 1 mated to deletion line 2, with parental lines and grandparental lines, along with uninjected 310x510 as controls. Lower gel section shows PCR amplification of TEBP-β as loading control. D) Quantitative PCR on the F1 from ctg14335.0 deletion line 1 mated to deletion line 2 surveyed across various regions of the contig14335.0 nanochromosome. E) Diagram of the various crosses involved generating the progeny involved in panels F and G. The colors from the diagram correspond to the colors in the following panel G. F) PCR amplification from genomic DNA harvested from populations of ctg14335.0 deletion line 1 backcrossed to wt strain JRB510 for up to 2 generations (labeled F1 and F2) with parental lines and grandparental lines along with uninjected 310x510 as controls. Lower gel section shows PCR amplification of TEBP-β as loading control. G) Quantitative PCR on genomic DNA used in panel A across multiple locations of ctg14335.0, together with an unrelated locus, TEBP-α. Arrows represent undetectable levels at the respective loci, with arrow color corresponding to the legend. Relative copy levels were determined by setting the levels of JRB310xJRB510 F1 to 1. H) Diagram of the various crosses involved generating the progeny in panels I and J. The colors from the diagram correspond to the colors in the following panel J. I) PCR amplification of genomic DNA harvested from clonal isolates and populations (labeled respectively) of the progeny of ctg14335.0 deletion line 2 backcrossed to JRB510, with parental and grandparental lines, along with uninjected JRB310xJRB510 as controls. Lower gel section shows PCR amplification of TEBP-β as loading control. J) Quantitative PCR on F1 genomic DNA from ctg14335.0 deletion line 2 across various regions of contig14335.0.

Cell lines containing the deletion of Contig14335.0 were mated to each other or backcrossed to the parental strain JRB510. The Contig14335.0 deletion lines mated to each other had a low survival rate, while the backcrosses showed no apparent survival defect. Both the deletion lines crossed to each other (Figure 4 panels C and D) and Contig14335.0 deletion line 1 backcrossed to JRB510 (Figure 4 panels F and G) showed no recovery of the deleted chromosome. DNA from backcrosses of Contig14335.0 deletion line 2 mated to JRB510 could amplify the full-length version of the deleted chromosome on a population level (Figure 4I).

To assess how common recovery was in the backcrossed progeny of Contig14335.0 deletion line 2 mated to JRB510, clonal lines were singled out. Nine such cell lines (out of 9) displayed no recovery of the full-length chromosome (Figure 4I), suggesting that recovery was infrequent. Quantitative PCR analysis of the backcrossed cells revealed an abundant center fragment derived from the deleted chromosome, with copy number at near wild type levels, as also seen in the parental Contig14335.0 deletion line 2 (Figure 4J). To test whether the internal telomere addition site in the parental deletion is inherited in the offspring, telomeric PCR and Sanger sequencing were performed (Supplementary figure 6). This confirmed that the highly abundant remnant in the F1 progeny has the same telomeric addition sites as those identified in the parental line, demonstrating that deletion of Contig14335.0 epigenetically transfers with incomplete penetrance to the subsequent generation, including the inheritance of a truncated telomere-containing chromosome fragment.

Previous experiments involving programmed alterations to macronuclear chromosome structure demonstrated the gradual reversion to the wild type genotype, *i.e.*, dilution of the programed alteration, after multiple sexual generations (Fang *et al.* 2012, Nowacki *et al.* 2008). We tested the recovery of the deleted chromosome after an additional sexual generation by mating the progeny of the Contig14335.0 deletion line 1 that had been backcrossed to JRB510 in a second backcross to JRB510. The resulting F2 cells still show no recovery of the targeted chromosome, as assessed by conventional PCR and qPCR (Figure 4 panels F and G). Inheritance of the Contig14335.0 deletion across multiple generations suggests a strong penetrance and dominant influence of the chromosome deletion in *Oxytricha*.

## Discussion

### Injection of synthetic chromosomes into mating cells results in deletion

Microinjection of a DNA template into *Oxytricha* cells during genome rearrangement can result in the deletion of the homologous sequence in the next generation macronucleus. This effect has been observed in many cases, implying that it is a general phenomenon. Strong sequence similarity is essential to target chromosome deletion, but the effect is robust to minor sequence variation, such as small deletions. Contig16116.0 and Contig20822.0 chromosome constructs containing small deletions, respectively 28 and 37 nucleotides, were still able to induce chromosome deletion. Surprisingly, whole genome sequencing revealed no significant off-target effects on nanochromosomes containing regions with high similarity to the deleted nanochromosomes (supplementary table 1). This suggests that target recognition occurs at a level above local similarity. We have not determined the precise level of similarity that is sufficient to cause the deletion effect in the subsequent generation.

Feeding *Oxytricha* cells *E. coli* expressing dsRNA from a plasmid containing a nanochromosome fragment previously resulted in failed rearrangement of the targeted chromosome (Nowacki *et al.* 2008). It is possible that the injected DNA in the current experiments may be acting in the same pathway as the previous RNAi feeding experiments. However, previous experiments that used DNA injection resulted in notably different results. In Nowacki *et al.* (2008) and (2010), injection at 1 to 3 hr post-pairing (slightly earlier than the 3 to 5 hr injection timepoint in the present study) of an entire synthetic nanochromosome, including telomere overhangs, resulted not in chromosome deletion but instead in *increased* chromosome copy number in the population of cells examined. The difference in these prior results from the current study may be due to subtleties in the timing of injection, or to differences between average effects on a population of cells (Nowacki *et al.* 2010) *vs.* individually isolated and cloned cells. Hence, the presence of a limited number of cells containing deletions may have been overlooked in Nowacki *et al.* (2008, 2010) because we observe deletions at a rate of ∼10% of injected cells, which would be missed in populations, without individual screening.

### Comparison to other DNA deletion phenomena in ciliates

While programmed DNA deletion is now observed in both *Oxytricha* and *Paramecium*, the apparent substrate requirements are different. On a structural level, the deletion in *Paramecium* depends on the presence of a truncated gene product in order to generate RNAi against the gene. This is not the case in *Oxytricha* as the injection of a full length nanochromosome results in its deletion from the next generation. Additional evidence supporting the conclusion that *Oxytricha* deletion is programmed via a separate mechanism is the ability to delete essential, early conjugation genes (Otiwi1 and AL1), given that silencing in injected cells would cause lethality immediately after injection. The apparent mechanistic differences between deletion in *Paramecium* and *Oxytricha* implicate an alternative model for deletion in *Oxytricha*.

In addition, the ciliate *Tetrahymena thermophila* has recently been reported to have endogenous programmed deletion of macronuclear chromosomes. 50 minichromosomes are transiently maintained and then lost after six generations of vegetative growth post-sexual development (Lin *et al.* 2016, Feng *et al.* 2017). Interestingly, these minichromosomes encode post-conjugation development specific genes, suggesting the possibility of programmed elimination from the macronucleus as a mechanism for gene regulation. While the phenomenon of macronuclear chromosome deletion that we report in *Oxytricha* is induced via the injection of an artificial chromosome, it bears similarity to the observed elimination of whole chromosomes in *Tetrahymena* as a mechanism to restrict gene expression to early macronuclear development.

### Implications of epigenetic inheritance of the deletions

The epigenetic inheritance shows different effects in the two cases examined, which seem to correlate with the fold-reduction in the parental deletion. In the case of Contig14335.0, deletion has a dominant effect, with the deletion propagated to the subsequent generation after parental backcross. In case of the Contig18510.0 deletion, the nanochromosome was recovered in the subsequent generation after parental backcross. One of the apparent variables between the two chromosome deletions is efficiency. The established Contig14335.0 deletion lines have strong copy number reduction, based on qPCR and genomic sequencing, whereas the Contig18510.0 deletion lines display only moderate reduction in copy number (∼six-fold decrease) based on qPCR results compared to wild type cells. The dominance of the deletion phenotype could be dependent on the strength in the parental deletion line. This dominant deletion inheritance is similar to the dominant inheritance of IES retention (Fang *et al.* 2012), where a normally deleted sequence is retained by progeny even when crossed to wild type cells.

The dominant inheritance of the chromosome deletion is surprising in the case of contig14335.0, based on the current model of early genome rearrangement. In *Oxytricha*, the piRNAs in association with Otiwi1 guide retention, while RNA templates help program rearrangement. Neither of these systems can easily explain the dominant inheritance of the chromosome deletion of contig14335.0. These results suggest that in addition to these known guiding RNA molecules, there are epigenetic elements that guide deletions. Hence, the deletion lines provide novel insight into the genome rearrangement process and can be used to further investigate the subtractive mechanisms at play.

### Model for the deletion phenomenon

We show that the injected template has an influence on the subsequent macronuclear generation, likely by interacting with one of the *Oxytricha* RNA pathways that guide rearrangement, and perhaps transcription of the injected templates could interfere with this pathway. We did not investigate transcription of the injected DNA, however, due to limited amount of material and the relative low efficiency of deletion (∼10% of injected cell lines result in chromosome deletion). As an alternative hypothesis to transcription of the injected product triggering DNA deletion, the injected DNA might inhibit or interfere directly with *Oxytricha*’s genome rearrangement pathway. In this second view, the injected DNA could inhibit the marking of native sequences for retention in the newly developing macronucleus, perhaps via soaking or sequestering of piRNA or RNA templates similar to the targeted sequence. In this model, the injected DNA would act as a sponge for RNA templates or piRNAs, leaving an inadequate level of RNA to target the native sequence for retention.

### Relevance of chromosome deletion

Targeted deletion of chromosomes has been previously demonstrated in other model systems through the use of various genetic tools (Matsumura *et al.* 2007, Li *et al.* 2012). Recently, targeted chromosome deletion has been generated in mice and human cell lines (Adikusuma *et al.* 2017, Zuo *et al.* 2017) through use of CRISPR/Cas9 and induction of targeted double stranded breaks. Chromosome deletion in *Oxytricha* offers an alternative demonstration of programmed chromosome deletion and emphasizes the diversity of genome rearrangement pathways in *Oxytricha*.

The heavy reduction in copy number of the targeted chromosome and their encoded gene(s) opens new avenues for generating somatic knockdowns or knockout strains. Moreover, despite the low rate of chromosome loss, it is still a viable method for generating mutant strains, because several dozen injected cells can be screened (even one 24-well plate can be reasonably expected to yield two deletion lines), and the resulting strains can be stably maintained and propagated for use in future experiments. Gene knockdown by IES retention (Fang *et al.* 2012; Khurana *et al.* 2018) suffers from a variety of sequence constraints, in particular requiring the IES to be near the amino-terminus of the target encoded protein in order to disrupt its open reading frame. The chromosome deletion strategy that we describe here is free of this limitation, thus allowing the functional analysis of a wider range of target genes. In addition, the chromosomal deletion approach offers a simple and more potent way to knock out target genes, compared to programmed IES retention.

## Appendix

### Primer list:

#### Chromosome creation primers

##### AL1:

5′ end Fw:

ATG AGA GTT TGT GAA AAA TTA AGT TTG TTG AGG TTA CTT TTG ATT GGT TAA TTA G

3′ end Rev:

TAT ATT AAA TAT CAA GAA AAA GTA AAA AGA CAG TAA AAA TAT ATA TAT ATT AAA TGC ATG

Deletion generation overlap extension Fw:

CTA CGC CCG TCC TTG TTC TCA AAA TCT CTC TCA TGG GTG GTC TGT TTT CTC TGT TTA TG

Deletion generation overlap extension Rev:

CAT AAA CAG AGA AAA CAG ACC ACC CAT GAG AGA GAT TTT GAG AAC AAG GAC GGG CG

5′ telomere addition Fw:

CCC CAA AAC CCC AAA ACC CCA TGA GAG TTT GTG AAA AAT TAA GTT TG

3′ telomere addition Rev:

CCC CAA AAC CCC AAA ACC CCT ATA TTA AAT ATC AAG AAA AAG TAA AAA GAC AG

##### Otiwi1:

5′ end Fw:

ATG AGT ATA ATT TGA ATT GTT GTA AAG AGA GTT TCC ATT TGA TTG

3′ end Rev:

GAT TAA TGG ATG GAA GAG ATA GAA GTA AAT TAG AGA TGA TTA ATT TAA TAT AAT TAT AAA

Deletion generation overlap extension Fw:

GGA ATC CGC TTT GAC ACC AAG CTT TCC CAA GGA CAA GAG CTC AAG TTG TTC TCC

Deletion generation overlap extension Rev:

GGA GAA CAA CTT GAG CTC TTG TCC TTG GGA AAG CTT GGT GTC AAA GCG GAT TCC

5′ telomere addition Fw:

CCC CAA AAC CCC AAA ACC CCA TGA GTA TAA TTT GAA TTG TTG TAA AGA GAG TTT CC

3′ telomere addition Rev:

CCC CAA AAC CCC AAA ACC CCA AAA GAT TAA T

##### Contig14335.0:

5′ telomere Fw:

CCC CAA AAC CCC AAA ACC CCG TAT TGG AAT TAT AAA CAA ATA TTA TGA TAG

3′ telomere Rev:

CCC CAA AAC CCC AAA ACC CCT TCC TGT ATA TTT CGA ATT TAA TCA G

##### Contig18510.0:

5′ telomere Fw:

CCC CAA AAC CCC AAA ACC CCA TTA ATT ATA TTG TTT TTG ACA TTG TAA AAT G

3′ telomere Rev:

CCC CAA AAC CCC AAA ACC CCA AGG AGT GTT TCA GAA ATA GAA AAA TTC

##### Contig17155.0:

5′ telomere Fw:

CCC CAA AAC CCC AAA ACC CCA TTA GAG ATA TAA CCA GAA TAT TTT ATG GG

3′ telomere Rev:

CCC CAA AAC CCC AAA ACC CCA TGG TTT GGA ATT TGA AAT CGT GAA GCA ATT AAT AT

##### Contig9679.0:

5′ telomere Fw:

CCC CAA AAC CCC AAA ACC CCA AGA ATT AAC TAT TGA AGT TGA TAA GTA AA

3′ telomere Rev:

CCC CAA AAC CCC AAA ACC CC T AAG TCT ATA TAA AAG TAT TGT TTT AAA AGT A

#### Detection primers

##### AL1:

Fw:

ATG AGA GTT TGT GAA AAA TTA AGT TTG TTG AGG TTA CTT TTG ATT GGT TAA TTA G

Rev:

GAG AGC ACA ACT ACA CTT TGC CTC ACA CTC

##### Otiwi1:

Same as Otiwi1 5′ end Fw and 3′ end Rev primers

##### Conttg14335:

Fw:

GAT AGA ATT GAA AGT AAA TTA GCT GTA AGG

Rev:

GAT CAA GCG ATC TAG CAA GGA G

##### Contig18510.0:

Fw:

TAA CAT AGT CCA GCA TTA CTA AAA TGA TG

Rev:

CTT AAA TGA GTT AAT ATA GAT CCA ACT CG

##### Contig17155.0:

Fw:

AAC TTA GCG GAA AAG TAA AAG CAA TAA TG

Rev:

TGA ACA TTA TCT TTC TTA AGC TGT TAC AAA AC

##### Contig9679.0:

Fw:

ATT GTA GTT ACT GTA TGC TGC TGG

Rev:

TGT CTT CTG TCC ATA CCT TGA AGG

##### TEBP-β (Contig22260.0):

Fw:

GAG CAA ATC ACA ACA AGT TCA ACA ACA AAG CG

Rev:

GCT CAT TTC TTG GTT GAT CTC TTT GAG GCC

##### Telomere detection:

AP12C4A4 (1^st^ round):

GTA ATA CGA CTC ACT ATA GGG CAC GCG TGG TCG ACG GCC CGG GCT GGT CCC CAA AAC CCC AAA ACC CCA AAA

Contig14335.0 telomeric fragment Rev (1^st^ round):

CAC TTA TGA TAA GCA TAT CAA TCG GGG CTC

AP2 (2^nd^ round):

ACT ATA GGG CAC GCG TGG T

Contig14335.0 telomeric fragment nested Rev (2^nd^ round):

GGA TTA CTG GTA CTG TAG AAA GAA GGA ATG

#### Southern primers

##### AL1 3′ Southern probe:

Fw:

ATG AGA CCA TCA ACA GAG ACT ATC CCT AAC

Rev:

CTC TTG TCT CTC CTT CTT GAA TCG AGG GTG

##### TEBP-β:

Same as TEBP-β detection chromosome

#### qPCR primers

##### Mito Cox1:

qPCR Fw:

GCC GTG TTT ACG CTT ATT TAC A

qPCR Rev:

CGT CTA GGC ATA CCA GCA TAT C

##### AL1 (Contig20822.0):

5′ qPCR Fw:

AAA GTC TGG TTC TCG TGG C

5′ qPCR Rev:

TCT GAA GCT TGT CCT TGG ATG

3′ qPCR Fw:

GAC AAG AGA CTA GCA GAC ACC

3′ qPCR Rev:

ACT ACA CTT TGC CTC ACA CTC

##### Otiwi1 (Contig16116.0):

5′ qPCR Fw:

TTT AAC AAG AAC AGA AAA GCA TTC AAG

5′ qPCR Rev:

TGG ATG GTG AAT CAA ACT CGA G

Mid qPCR Fw:

CCT GAG ATT TGA GAC CAT CGG

Mid qPCR Rev:

AGT CTA GCA TCA AAT CCA GGC

3′ qPCR Fw:

GAG ACC AAG GAG CCA ACC

3′ qPCR Rev:

TCC AAT TAG TAG TTG CCA TGA GAG

##### Contig14335.0:

5′ qPCR Fw:

TCT CTC ATG AAC GGT TAG AGT TTA G

5′ qPCR Rev:

GGA TGA GTT TAT CGA TTC TGC ATT AG

Mid qPCR Fw:

CCA ATA CTC GAA GTC CTC CTT G

Mid qPCR Rev:

GGT AAT GGA TTA CTG GTA CTG TAG AA

3′ qPCR Fw:

GCT CTC GAG CTC ATC TGA TTC

3′ qPCR Rev:

TCT CCA CTT AAT GAC GAG TAC TTA TAC

##### Contig18510.0:

5′ qPCR Fw:

CTG TCA TAT TAA CAT AGT CCA GCA TTA C

5′ qPCR Rev:

ATC CGT ATT CCT GTC TTC CAT TAG

Mid qPCR Fw:

AGT ACT TCT CTC AAA CAG GAT AGA AC

Mid qPCR Rev:

AGA TAT TGG AGT CTC TCT TTG TGT T

3′ qPCR Fw:

TTC ACA ACA GCC TAA GAA GTA TCT

3′ qPCR Rev:

CAT TTG TAT CAT TCA ATG GCA CTC T

##### TEBP-α (Contig22209.0):

qPCR Fw:

CCA CAG AGC CAC CAT CAG ACT TTA

qPCR Rev:

GAG AAT GGT ACG AGA TCG CTA GTA GC
